# Re-thinking all-cause COVID-19 hospitalizations as a surrogate measure for severe illness in observational surveillance studies

**DOI:** 10.1038/s41598-024-61244-7

**Published:** 2024-06-24

**Authors:** J. Daniel Kelly, Samuel Leonard, W. John Boscardin, Katherine J. Hoggatt, Emily N. Lum, Charles C. Austin, Amy L. Byers, Phyllis C. Tien, Dawn M. Bravata, Salomeh Keyhani

**Affiliations:** 1 San Francisco VA Medical Center, 4150 Clement Street 111A1, San Francisco, CA 94121 USA; 2 Department of Medicine, University of California, San Francisco (UCSF), San Francisco, CA USA; 3 Department of Epidemiology and Biostatistics, UCSF, San Francisco, CA USA; 4 F.I. Proctor Foundation, UCSF, San Francisco, CA USA; 5 Department of Veterans Affairs (VA) Health Services and Development (HSR&D) Center for Health Information and Communication (CHIC), Department of Medicine, Richard L. Roudebush VA Medical Center, Indianapolis, IN USA; 6 Department of Psychiatry and Behavioral Sciences, UCSF, San Francisco, CA USA; 7 Department of Medicine, Indiana University School of Medicine, Indianapolis, IN USA; 8 Regenstrief Institute, Indianapolis, IN USA

**Keywords:** Infection, Infectious diseases

## Abstract

All-cause COVID-19 hospitalization ≤ 30 days of infection is a common outcome for severe illness in observational/surveillance studies. Milder COVID-19 disease and COVID-19-specific measurements calls for an evaluation of this endpoint. This was a descriptive, retrospective cohort study of adults ≥ 18 who were established in primary care at Veteran Health Administration (VHA) facilities. The outcome was hospitalization within 30 days of a laboratory-confirmed, symptomatic SARS-CoV-2 infection. Between December 15, 2021 and May 1, 2022, a simple random sample of all VA facilities, excluding Puerto Rico or Philippines, was drawn to identify these hospitalized cases and determine whether hospitalization was due to COVID-19-specific causes. A chart review was conducted to record the inpatient clinical team’s diagnosis and whether the inpatient team classified the diagnosis as COVID-19 related or not. These data were used to classify hospitalizations as either due to COVID-19-specific causes (direct manifestations of SARS-CoV-2 infection) or non-COVID-19-specific hospitalizations (incidental SARS-CoV-2 infection), A simple random sample of 9966 (12.3%) all-cause hospitalizations (95% CI: 12.1%, 12.5%) was used to select 300 representative patients. Of these, 226/300 (75.3%) were determined to be COVID-19-specific. COVID-19 pneumonia was most common (147/226, 65.0%). The highest proportion of COVID-19-specific hospitalizations occurred among unvaccinated (85.0%), followed by vaccinated but not boosted (73.7%) and boosted (59.4%) (*p* < 0.001). The proportion of non-COVID-19-specific hospitalizations was higher in the later period (15–30 days: 55.0%) than the early (0–15 days: 22.5%) (*p* = 0.003). This study supports the outcome of COVID-19-specific hospitalization instead of all-cause hospitalization in observational studies. The earlier outcome period (0–15 days) was less susceptible to potential measurement bias.

## Introduction

Observational, surveillance studies rely on readily available data from electronic health records, insurance databases, or other sources to assess the effectiveness of COVID-19 vaccines or therapeutics through a target trial emulation approach^1–5^. This approach uses all-cause hospitalization after symptomatic COVID-19 as a proxy for COVID-19-related hospitalization with the intent to measure severe illness. In contrast, clinical trials of COVID-19 vaccines and therapeutics have the intensive resources to directly measure COVID-19-related hospitalization^6–8^. The U.S. Veterans Health Administration (VHA) has access to detailed, patient-level data so that chart reviews can be conducted, offering the ability to evaluate all-cause COVID-19 hospitalization for mismeasurement biases.

In an era of vaccines, increasing natural immunity, and viral evolution, it is important to re-evaluate all-cause COVID-19 hospitalization as a useful surrogate outcome in observational studies because COVID-19 disease has been associated with less severe illness and complications, including pneumonia, acute myocardial infarction, and ischemic stroke after infection^9^. Without the late occurrence of cytokine storms, progression to severe illness typically occurs over a shorter period, suggesting that measurement of all-cause COVID-19 hospitalization over a 30-day period may be more likely to detect hospitalizations unrelated than related to COVID-19. At the same time, COVID-19 remains a relatively common occurrence^10^ and infected, symptomatic individuals can be hospitalized for many reasons that may be unrelated to COVID-19 disease (incidental SARS-CoV-2 infections)^10^.

During a period with a predominance of Omicron SARS-CoV-2 variants, this study drew a random sample of all-cause COVID-19 hospitalizations among a national cohort of U.S. Veterans. Reasons for hospitalization documented by the inpatient care team in medical charts were abstracted and used to determine whether the hospitalization was due to a COVID-19-specific cause.

## Methods

### Ethics statement

The institutional review board of the University of California, San Francisco, approved this study and waived requirement for patient consent, due to the retrospective nature of the study. All methods were performed in accordance with the relevant guidelines and regulations. We followed the Strengthening the Reporting of Observational Studies in Epidemiology (STROBE) reporting guideline.

### Study design, data sources, and participants

We conducted a retrospective cohort study using chart review to describe the proportion of all-cause hospitalizations due to COVID-19-specific or non-COVID-19-specific causes among hospitalized patients with laboratory-confirmed, symptomatic SARS-CoV-2 infection. The cohort included adults aged 18 or older who were established at the U.S. VHA facilities. Exclusion criteria included (1) use of nursing home or hospice care services within the 2 years prior to a COVID-19 diagnosis, and (2) hospitalization in Puerto Rico or the Philippines. We used VA Corporate Data Warehouse (CDW)^11^ and COVID-19 shared data resource^12^ to construct the cohort and identify patients who had laboratory-confirmed, symptomatic SARS-CoV-2 infection and were hospitalized within 30 days of infection. COVID-19 shared data resource was established to document symptomatic infections and hospitalizations occurring outside of VA facilities and integrate these data back into the VA health records. A random sample of hospitalizations was drawn for chart review between December 15, 2021 and May 1, 2022. Thirty percent of these charts were reviewed in duplicate.

### Measurements

When reviewing charts, study staff recorded the inpatient clinical team’s diagnosis and whether the inpatient team classified the diagnosis as COVID-19 related or not. Diagnoses related to COVID-19 were defined as COVID-19-specific (direct manifestations of SARS-CoV-2 infection), and those unrelated to COVID-19 were defined as non-COVID-19-specific causes (incidental SARS-CoV-2 infection).

Any hospitalization with unclear labeling and/or disagreement amongst the chart abstractors about the diagnosis being related to COVID-19 was flagged for review by three clinicians (JDK, SK, DMB) and consensus was reached via discussion. An example of unclear labeling in a person with symptomatic SARS-CoV-2 infection was pneumonia, either due to bacterial and viral pathogen, so unless these were witnessed aspiration events, the pneumonia was classified as COVID-19-related since bacterial and viral infections were radiologically indistinguishable. The study team did not overrule any diagnoses or etiological statements made by the clinical team.

Covariates used to describe the cohort were extracted from the VA Corporate Data Warehouse. Those with high-risk comorbid and immunocompromising conditions were classified using U.S. Centers for Disease Prevention and Control definitions^10,13^.

### Statistical analysis

The cumulative incidence of all-cause hospitalization was described in the overall cohort and sub-groups stratified by vaccination status (unvaccinated [no vaccine dose], vaccinated but not boosted [received primary series], boosted [received all recommended doses]) and time since infection (0–15 days, 16–30 days). In the random sample, reasons for hospitalization as determined by the abstraction team’s chart review were categorized as COVID-19-specific causes, and non-COVID-19-specific causes. Descriptive statistics with Chi-squared tests were used to compare the proportion of all-cause and cause-specific hospitalizations among those related and not related to COVID-19, stratified by vaccination status and by time since COVID-19 infection. A 2-tailed p < 0.05 was considered significant. Analyses were conducted in R version 1.2.5019.

## Results

**Figure 1:**
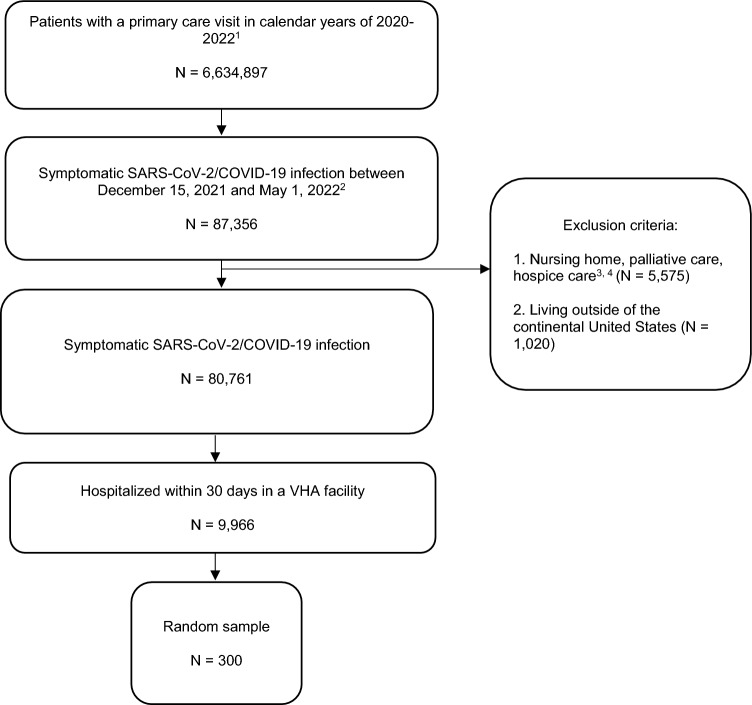
Cohort selection in U.S. cohort of patients receiving care at Veterans Health Administration (VHA) facilities during a period of Omicron variant predominance and were hospitalized within 30 days of a symptomatic SARS-CoV-2 infection.

Among 6,634,897 patients receiving care at VHA facilities, 80,761 had laboratory-confirmed, symptomatic SARS-CoV-2 infection (121.7 events per 10,000 persons; 95% CI: 120.9, 122.6). Among these infected patients, there were 9966 (12.3%) all-cause hospitalizations (95% CI: 12.1%, 12.5%). A simple random sample of this hospitalized population was used to select 300 patients who were representative of the national cohort (Fig. 1). Among these 300 hospitalized patients, 196 (65.3%) were aged 65 or older and 17 (5.7%) were female; 198 (66.0%) had high-risk comorbid conditions and 67 (22.3%) had an immunocompromising condition. See Table 1 for additional characteristics.

**Table 1:** Characteristics of overall hospitalized cohort (N = 300).

	Overall cohort, N = 300
Male	283 (94.3)
Female	17 (5.7)
Age, 18–44	31 (10.3)
Age, 45–64	73 (24.3)
Age, 65–74	109 (36.3)
Age, 75–84	60 (20.0)
Age, 85 or older	27 (9.0)
American Indian or Alaska Native	3 (1.0)
Asian	2 (0.7)
Black or African American	74 (24.7)
White	202 (67.3)
Native Hawaiian or other Pacific Islander	2 (0.7)
More than one race	2 (0.7)
Unknown	15 (5.0)
Hispanic or Latino ethnicity (regardless of race)	20 (6.7)
Married	131 (43.7)
Urban	220 (73.3)
Rural	64 (21.3)
Highly rural	7 (2.3)
Unknown	9 (3.0)
Prior history of COVID-19	8 (2.7)
Hypertension	213 (71.0)
CHF	56 (18.7)
IHD	78 (26.0)
Diabetes	115 (38.3)
Stroke TIA	18 (6.0)
COPD Bronchiectasis	63 (21.0)
Cirrhosis	10 (3.3)
Dementia	27 (9.0)
Dialysis	12 (4.0)
Cancer-solid organ	20 (6.7)
Cancer-lymphoma leukemia	11 (3.7)
Cancer-other	6 (2.0)
Current smoker	71 (23.7)
Alcohol use disorder	45 (15.0)
Substance use disorder	40 (13.3)
Housing problems	34 (11.3)
BMI, < 18.5	3 (1.0)
BMI, 18.5–24.9	52 (17.3)
BMI, 25–29.9	96 (32.0)
BMI, > = 30	111 (37.0)
Unknown BMI	38 (12.7)
No Chronic kidney disease	204 (68.0)
Chronic kidney disease	76 (25.3)
Severe chronic kidney disease	20 (6.7)
VA district, Continental	49 (16.3)
VA district, Midwest	51 (17.0)
VA district, North Atlantic	69 (23.0)
VA district, Pacific	63 (21.0)
VA district, Southeast	68 (22.7)
Hospitalization 0–15 days post infection	280 (93.3)
Unvaccinated	127 (42.3)
Vaccinated-not boosted	99 (33.0)
Boosted	69 (23.0)
Vaccinated Status unclear	5 (1.7)
Age > = 65 and no high-risk conditions	21 (7.0)
High-risk co-morbid conditions (not immunocompromised),	198 (66.0)
Immunocompromised	67 (22.3)

Among all-cause hospitalizations, 226/300 (75.3%) were determined to be COVID-19-specific. The most common causes of the 226 COVID-19-specific hospitalizations were as follows: COVID-19 pneumonia, 147 (65.0%); cardiovascular events (e.g., myocarditis, new onset atrial fibrillation), 23 (10.2%); weakness/falls, 12 (5.3%); COPD/asthma exacerbation, 8 (3.5%); neurocognitive disorders (e.g., encephalopathy), 8 (3.5%); and gastrointestinal (GI) illness (e.g., diarrhea), 10 (4.0%). The remaining hospitalizations (74/300, 24.7%) were non-COVID-19-specific. The most common causes of the 74 non-COVID-19-specific hospitalizations were as follows: GI illness (e.g., recurrent GI bleeding, abdominal pain from constipation), 17 (23.0%); mental illness or substance abuse (e.g., alcohol withdrawal), 16 (21.6%); non-COVID infectious disease (e.g., urinary tract infection), 15 (20.3%); cardiovascular events (e.g., hypertensive emergency off blood pressure medications), 6 (8.1%); and pain syndrome, 6 (8.1%). See Table 2 for other diagnoses classified as COVID-specific or non-COVID-19-specific hospitalizations. See Supplement for descriptions of these diagnoses during the hospitalization and whether the case was classified as COVID-19-specific or non-COVID-19-specific.

**Table 2:** Classification of all-cause hospitalizations as COVID-19-specific hospitalizations (N = 247) or non-COVID-19-specific hospitalizations (N = 73).

	Total	By vaccination status					By time elapsed between breakthrough infection and admission		
		Boosted	Vaccinated, not boosted	Not vaccinated	Status unclear	*p*-value for COVID-specific versus non-specific*	Hospitalized 0–15 days	Hospitalized 16–30 days	*p*-value for COVID-specific versus non-specific
Overall	300	69	99	127	5		280	20	
COVID-19-specific hospitalizations									
All	226 (75.3)	41 (59.4)	73 (73.7)	108 (85.0)	4 (80.0)	< 0.001	217 (77.5)	9 (45)	0.003
COVID-19 pneumonia	147 (65.0)	26 (63.4)	40 (54.8)	79 (73.1)	2 (50.0)		145 (66.8)	2 (22.2)	
Cardiovascular events	23 (10.2)	3 (7.3)	10 (13.7)	9 (8.3)	1 (25.0)		22 (10.1)	1 (11.1)	
Weakness/falls	12 (5.3)	2 (4.9)	6 (8.2)	3 (2.8)	1 (25.0)		11 (5.1)	1 (11.1)	
COPD/asthma exacerbation	8 (3.5)	4 (9.8)	0 (0.0)	4 (3.7)	0 (0.0)		7 (3.2)	1 (11.1)	
Neurocognitive disorders	8 (3.5)	1 (2.4)	4 (5.5)	3 (2.8)	0 (0.0)		7 (3.2)	1 (11.1)	
Gastrointestinal illness	8 (3.5)	0 (0.0)	4 (5.5)	4 (3.7)	0 (0.0)		7 (3.2)	1 (11.1)	
Other	6 (2.7)	2 (4.9)	4 (5.5)	0 (0.0)	0 (0.0)		6 (2.8)	0 (0.0)	
Thromboembolic events	4 (1.8)	1 (2.4)	2 (2.7)	1 (0.9)	0 (0.0)		4 (1.8)	0 (0.0)	
Isolation/observation for COVID 19	4 (1.8)	1 (2.4)	0 (0.0)	3 (2.8)	0 (0.0)		4 (1.8)	0 (0.0)	
Kidney disease	3 (1.3)	1 (2.4)	1 (1.4)	1 (0.9)	0 (0.0)		2 (0.9)	1 (11.1)	
Non-COVID infectious disease	3 (1.3)	0 (0.0)	2 (2.7)	1 (0.9)	0 (0.0)		2 (0.9)	1 (11.1)	
Non-COVID-19-specific hospitalizations									
All	74 (24.7)	28 (40.6)	26 (26.3)	19 (15.0)	1 (20.0)		63 (22.5)	11 (55.0)	
GI illness	17 (23.0)	9 (32.1)	5 (19.2)	3 (15.8)	0 (0.0)		13 (20.6)	4 (36.4)	
Mental illness or substance abuse	16 (21.6)	3 (10.7)	6 (23.1)	7 (36.8)	0 (0.0)		13 (20.6)	3 (27.3)	
Non-COVID infectious disease	15 (20.3)	8 (28.6)	4 (15.4)	2 (10.5)	1 (100.0)		14 (22.2)	1 (9.1)	
Cardiovascular events	6 (8.1)	2 (7.1)	2 (7.7)	2 (10.5)	0 (0.0)		6 (9.5)	0 (0.0)	
Pain syndrome	6 (8.1)	2 (7.1)	2 (7.7)	2 (10.5)	0 (0.0)		6 (9.5)	0 (0.0)	
Genitourinary illness	5 (6.8)	2 (7.1)	1 (3.8)	2 (10.5)	0 (0.0)		4 (6.3)	1 (9.1)	
Other	4 (5.4)	1 (3.6)	2 (7.7)	1 (5.3)	0 (0.0)		4 (6.3)	0 (0.0)	
Neurocognitive disorders	2 (2.7)	0 (0.0)	2 (7.7)	0 (0.0)	0 (0.0)		2 (3.2)	0 (0.0)	
Oncology	2 (2.7)	1 (3.6)	1 (3.8)	0 (0.0)	0 (0.0)		1 (1.6)	1 (9.1)	
Kidney disease	1 (1.4)	0 (0.0)	1 (3.8)	0 (0.0)	0 (0.0)		0 (0.0)	1 (9.1)	

*More details of these diagnoses are described in the Supplement.

The proportion of COVID-19-specific hospitalizations among all-cause hospitalizations varied by vaccination status. The highest proportion of COVID-19-specific hospitalizations occurred among the unvaccinated group (108/127, 85.0%), followed by the vaccinated but not boosted group (73/99, 73.7%) and boosted group (41/69, 59.4%) (*p* < 0.001). Stratified by time since infection (0–15 days, 15–30 days), the majority of all-cause hospitalizations (280, 93.3%) occurred within the first 15 days of infection. The proportion of non-COVID-19-specific hospitalizations was higher in the later period (15–30 days: 11/20, 55.0%) than the early period (0–15 days: 63/280, 22.5%) (*p* = 0.003) (Table 2).

## Discussion

In this cohort of patients hospitalized at VHA facilities 30 days after symptomatic SARS-CoV-2 infection, we evaluated the extent to which all-cause hospitalization within 30 days of symptomatic SARS-CoV-2 infection remains a useful outcome for severe COVID-19 illness in observational, surveillance studies. Consistent with the literature^14,15^, about one-quarter of all-cause hospitalizations were non-COVID-19-specific (incidental SARS-CoV-2 infection). A higher proportion of non-COVID-19-specific hospitalizations occurred among those who were boosted than unvaccinated and among those hospitalized 16–30 days instead of 0–15 days after infection. As the U.S. population has become highly immunized to COVID-19 either through vaccination or infection and as the clinical spectrum of illness has moderated^9^, continued use of all-cause hospitalization within 30 days of symptomatic SARS-CoV-2 infection as a study outcome should be approached with caution because of its trade-off between sensitivity and specificity.

Hospitalization within 15 days after symptomatic SARS-CoV-2 infection was a more specific measurement of COVID-19 hospitalization than within 30 days and may be less prone to measurement bias. Over time, the likelihood that SARS-CoV-2 infection results in a hospitalization due to COVID-19-specific causes decreases; thus we expect to observe more incidental SARS-CoV-2 infections during this later period (16–30 days). In addition, summary measures of COVID-19-specific hospitalization have yet to be validated. This study found hospitalization due to COVID-19 pneumonia accounted for most COVID-19-specific causes and can be identified with an ICD-10 code and chart review. To this end, most studies attempting to use a specific outcome have focused on the most common, cause-specific diagnoses such as pneumonia^10^, and to a lesser extent, arterial and venous thrombotic events^16^. A tradeoff of these more focused analyses has been the loss of sensitivity, which could slow down research on time-sensitive questions.

There are limitations of this study. First, we relied on the inpatient medical team’s assessment of the cause for hospitalization. In some cases, the team’s indicated diagnosis was clearly not related to COVID-19 (e.g., fracture, fall, pain, substance use) but in other cases distinguishing etiology of symptoms was more challenging (e.g., progression of kidney disease). Second, the study population was predominantly White men and included community-dwelling individuals living in the U.S. Third, it is likely that the proportion of all-cause hospitalization (12.3%) was an overestimate given the timeframe under investigation (large Omicron wave). Additional limitations of generalizability extend to Omicron variants beyond BA.1 and BA.2 and after receipt of the bivalent Omicron booster vaccine. Finally, during the observational study period, rapid antigen test became available, so the total number of cases was an underestimate.

Evidence from this study supports use of COVID-19-specific hospitalization in many scenarios instead of all-cause hospitalization in observational surveillance studies. Further, the earlier period (0–15 days) of the outcome was less susceptible to potential measurement bias in contrast to the later period (16–30 days). This report highlights the importance of re-evaluating clinical endpoints in an evolving landscape of viral variants and booster vaccine.

### Supplementary Information

Supplementary Information.

## Data Availability

The data that support the findings of this study are available from Veterans Health Administration, but restrictions apply to the availability of these data, which were used under license for the current study, and so are not publicly available. Data are however available from the authors upon reasonable request and with permission of the Veterans Health Administration. Please contact Dr. Dan Kelly (dan.kelly@ucsf.edu) to request information regarding access for data from this study. More information is available at https://www.virec.research.va.gov. Source data are provided with this paper.
