# Supplementary material for: Re-thinking all-cause COVID-19 hospitalizations as a surrogate measure for severe illness in observational surveillance studies
Source: Sci Rep. 2024 Jun 24;14:14555. doi: 10.1038/s41598-024-61244-7 (PMC11196634; doi:10.1038/s41598-024-61244-7)
Supplement: Supplementary file 1 — Supplementary Information. [file 41598_2024_61244_MOESM1_ESM.docx]

**Supplemental Material**

**Re-thinking all-cause COVID-19 hospitalizations as a surrogate measure for severe illness in observational surveillance studies**

**Authors:**

J. Daniel Kelly^1,2,3,4*^, MD, PhD

Samuel Leonard^1^, MS

W. John Boscardin^3^, PhD

Katherine J. Hoggatt^1,2^, PhD

Emily N. Lum^1^, MPH

Charles C. Austin^5^, MDiv

Amy L. Byers^1,2,6^, PhD, MPH

Phyllis C. Tien^1,2^, MD

Dawn M. Bravata^5,7,8^, MD

*Salomeh Keyhani^1,2^, MD

**Affiliations:**

1. San Francisco VA Medical Center, San Francisco, CA, USA;

2. Department of Medicine, University of California, San Francisco (UCSF), CA, USA;

3. Department of Epidemiology and Biostatistics, UCSF, CA, USA;

4. F.I. Proctor Foundation, UCSF, CA, USA;

5. Department of Veterans Affairs (VA) Health Services and Development (HSR&D) Center for Health Information and Communication (CHIC) and the Department of Medicine, Richard L. Roudebush VA Medical Center; Indianapolis, IN, USA;

Veterans Affairs Medical Center, Indianapolis, IN, USA;

6. Department of Psychiatry and Behavioral Sciences, UCSF, CA, USA;

7. Department of Medicine, Indiana University School of Medicine, Indianapolis, IN, USA;

8. Regenstrief Institute, Indianapolis, IN, USA.

eTable 1. Examples of COVID-19 specific hospitalizations and non-COVID-19 specific hospitalizations.

| **Outcome** | **Hospitalization Description** | **COVID-19-specific hospitalization** |
| --- | --- | --- |
| COVID-19 Pneumonia | Covid19 Pneumonia, CXR with groundglass opacities in the R lung field and hypoxia required 2L of NC, was weaned as tolerated. | Yes |
| COVID-19 Pneumonia | Acute Hypoxic Respiratory Failure 2ndary to Covid 19, Pt saturations improved from 80 to 95% on 4L NC | Yes |
| Cardiovascular event | Acute MI pt was taken for urgent coronary angiogram. COVID19 | Yes |
| Cardiovascular events | Acute on Chronic HFrEF, secondary to non-ischemic cardiomyopathy, stage D, Cardiogenic Shock, LV Apical Thrombus | No |
| Cardiovascular event | ED from Cardiology Clinic due to 1-2 weeks of crushing L sided chest pain that radiates to shoulders and L arm and worsens to 9/10 on exertion. Will closely monitor given COVID + and unvaccinated. | Yes |
| Cardiovascular event | Congestive Heart Failure, HTN, CAD Exacerbation | No |
| Weakness/falls | Admitted to telemetry for decompensated CHF and debility with frequent falls due to COVID | Yes |
| Weakness/falls | Generalized weakness and falls-likely secondary to sequelae of COVID 19 infection vs hypoglycemia. | Yes |
| COPD/asthma exacerbation | COPD exacerbation caused by Covid infection | Yes |
| COPD/asthma exacerbation | Acute on chronic resp failure due to AECOPD with hypoxia, COVID Positive in triple vaccinated with booster | Yes |
| Neurocognitive disorders | Presented with two months of acute on chronic cognitive decline, increased falls, and urinary incontinence, post COVID+ | Yes |
| Neurocognitive Disorders | Presented with altered mental status, poor oral intake and admitted with encephalopathy. | No |
| Neurocognitive disorders | Encephalopathy: acute on chronic from admission in the setting of COVID | Yes |
| Gastrointestinal illness | 1. COVID-19 Infection, 2. Cholelithiasis | Yes |
| Gastrointestinal illness | Covid-19+ on admission. Admitted for syncope and AKI, secondary to orthostatic hypotension and dehydration due to covid-related diarrhea | Yes |
| GI illness | Acute diarrhea in the setting of microscopic collagenous colitis. | No |
| GI illness | Reports up to 8 bowel movements per day over the weekend without symptoms associated with covid. | No |
| Genitourinary illness | One day history of blood in his urine but denies dysuria | No |
| Genitourinary illness | Management of UTI and delirium. | No |
| Other | Intermittent nonproductive cough and nasal congestion/drainage. Covid test came back positive today. | Yes |
| Other | PRINCIPAL DIAGNOSIS: Diabetes, symptomatic hypocalcemia | No |
| Other | Poorly controlled DMII, chronic pancreatitis, CAD, neuropathy, MDD, and PTSD who presents to the ED with a 1 day history of chest pain, abdominal pain, nausea, and vomiting. | No |
| Other | COVID, Fluid Overload, possible Heart Failure Preserved Ejection Fraction  #Emphysema  -s/p Remdesivir x2 days (given because pt was not vaccinated) | Yes |
| Thromboembolic events | Pulmonary embolism. (Present at admission). Dyspnea worsened over last day. Tested positive for COVID about 2 weeks ago. Symptoms started after that. | Yes |
| Thromboembolic events | Bilateral leg DVT and COVID PCR positive | Yes |
| Isolation/observation for COVID19 | Veteran transferred to hospital for isolation due to mildly symptomatic Covid 19. | Yes |
| Isolation/observation for COVID19 | Transferred to complete isolation, after which he can return to his transitional living. | Yes |
| Kidney disease | Acute Kidney injury, found to be COVID positive (NOT vaccinated) | Yes |
| Kidney disease | Shock - hypovolemic vs Septic, likely multifactorial; Hyponatremia with accompanying hypochloremia, Acute on chronic kidney injury | No |
| Kidney disease | Reason for Admission: ACUTE RENAL FAILURE and COVID+ | Yes |
| Non-COVID infectious disease | Sinusitis as noted above. | Yes |
| Mental illness or substance abuse | PRIMARY DIAGNOSIS: acute alcohol intoxication | No |
| Mental illness or substance abuse | Reported ongoing use of multiple substances and requested admission for detox and referral to treatment. | No |
| Non-COVID infectious disease | PRIMARY DIAGNOSIS FOR THIS ADMISSION: panniculitis, groin cellulitis and dermatitis | No |
| Non-COVID infectious disease | PRIMARY DIAGNOSIS-LLE cellulitis d/t cat scratch | No |
| Pain syndrome | L4/5 herniated disk who presented with worsening of right leg pain and numbness over the last several days. | No |
| Pain syndrome | Reason for Admission: right hand fracture | No |
| Oncology | Left orbital mass, Lambda light-chain multiple myeloma | No |
